# Supplementary material for: Modulation-free laser stabilization technique using integrated cavity-coupled Mach-Zehnder interferometer
Source: Nat Commun. 2024 Mar 1;15:1922. doi: 10.1038/s41467-024-46319-3 (PMC10907685; doi:10.1038/s41467-024-46319-3)
Supplement: Supplementary file 1 — Supplementary Information [file 41467_2024_46319_MOESM1_ESM.pdf]

Modulation-free laser stabilization technique using integrated cavity-coupled Mach-Zehnder interferometer

Mohamad Hossein Idjadi[1,*], Kwangwoong Kim[1], and Nicolas K. Fontaine[1]

[1]Nokia Bell Labs, 600 Mountain Ave, Murray Hill, NJ 07974, USA.

[*]Corresponding author: mohamad.idjadi@nokia-bell-labs.com

Supplementary Information

Supplementary Note 1: the error signal of the cavity-coupled MZI OFND

Supplementary Figure 1a shows the block diagram of a generalized cavity-coupled MZI optical frequency noise discriminator (OFND) architecture. Within this model, we consider a generalized transfer function of an optical frequency reference, $T(.)$. The electric field at the input of the OFND can be expressed as

$$E_{in}(t) = \sqrt{P_0}e^{j\omega t}, \tag{1}$$

where ω and P_0 are the instantaneous laser frequency and power, respectively. A Y-junction is used to split the power equally. The electric fields before the output coupler are

$$E_1(t) = \sqrt{\frac{P_0}{2}}T(\omega)e^{jwt}, \tag{2}$$

$$E_2(t) = \sqrt{\frac{P_0}{2}}e^{j\phi}e^{jwt}, \tag{3}$$

where $T(\omega)$ and ϕ are the complex transfer function of the optical frequency reference and the phase shift introduced by the thermal phase shifter at the bottom branch of MZI, respectively. A directional coupler combines the electric field. The electric fields at the coupler output are

$$E_{o1}(t) = \frac{1}{\sqrt{2}}(E_1(t) + jE_2(t)), \tag{4}$$

$$E_{o2}(t) = \frac{1}{\sqrt{2}}(E_2(t) + jE_1(t)). \tag{5}$$

The optical signal at the output of the MZI is photodetected using a balanced photodetector (BPD). The photocurrents i_1 and i_2 are calculated as

$$i_1(t) = R|E_{o1}(t)|^2, \tag{6}$$

$$i_2(t) = R|E_{o2}(t)|^2, \tag{7}$$

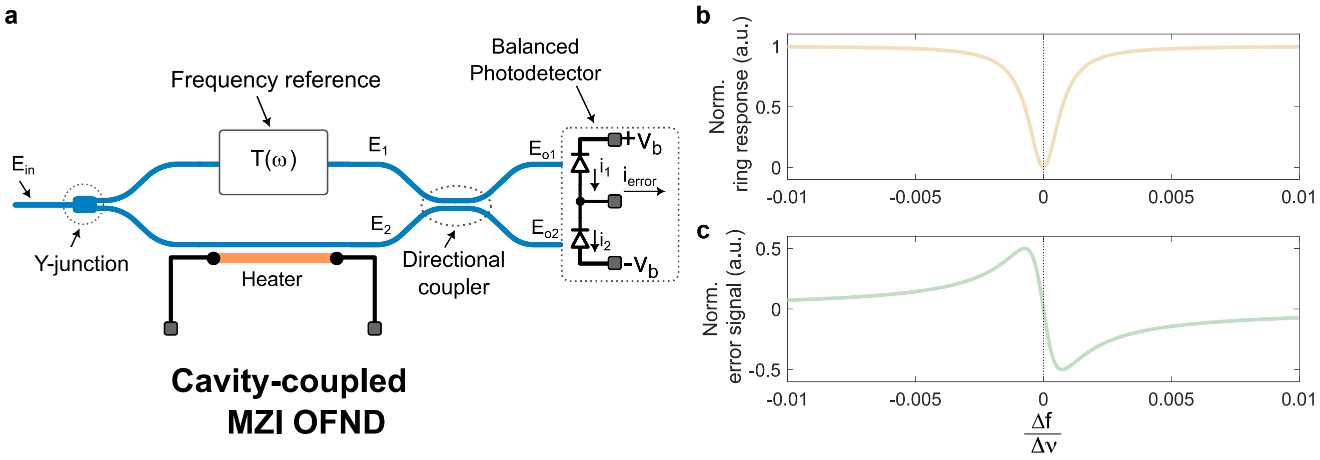

Supplementary Figure 1 | The cavity-coupled MZI OFND. a The block diagram of the proposed cavity-coupled MZI optical frequency noise discriminator (OFND) architecture. **b** The simulated ring resonator response with circumference of 1 mm and waveguide loss of 0.2 dB.cm^{-1}. **c** The asymmetric error signal. In this example, the normalized gain of the OFND is 1.7$\times$10^{-8} Hz^{-1}. Δf and $\Delta\nu$ are offset frequency compared to f_{ref} and the free-spectral range of the ring resonator, respectively.

where R is the responsivity of the photodetectors. Using Supplementary Equations (4) to (7), the error signal, $i_1(t) - i_2(t)$, can be written as

$$i_{error}(t) = 2R\Im\Bigg(E_1(t)E_2(t)^*\Bigg), \quad (8)$$

where $\Im(.)$ denotes the imaginary operator. Combining Supplementary Equations (2),(3), and (8), the error signal can be simplified to

$$i_{error}(\omega) = RP_0|T(\omega)| \times \sin(\psi(\omega) - \phi), \quad (9)$$

where $|T(\omega)|$ and $\psi(\omega)$ are the amplitude and phase of the optical reference transfer function at the frequency of ω. Please note that the frequency of laser, ω, can be written as

$$\omega(t) = \omega_0 + \delta\omega_n(t), \quad (10)$$

where ω_0 and $\delta\omega_n(t)$ are the nominal frequency and frequency noise of the laser, respectively. Supplementary Figure 1b, c show the numerical simulation of the frequency reference response and the asymmetric error signal, respectively. For numerical analysis purposes, we assume that the frequency reference is a critically coupled ring resonator with an approximately 80 GHz free-spectral range (FSR) (equivalent to a 1 mm circumference) and a Q-factor of around 3.3×10^6, corresponding to a propagation loss of approximately 0.2 dB.cm^{-1}. As shown in Supplementary Figure 1c, the error signal exhibits an asymmetric response around the frequency reference that can be used as a servo signal to lock the laser to the frequency reference.

Supplementary Note 2: comparative analysis of different OFND architectures

Numerous OFNDs have been proposed and demonstrated in the past, including the well-known PDH architecture[1] and the unbalanced MZI. Supplementary Figures 2 a-c show the block diagram of the PDH, an unbalanced MZI, and the proposed cavity-coupled MZI OFNDs, respectively[2]. In the PDH circuit, as shown in Supplementary Figure 2a, an electrical local oscillator modulates the incoming electric field using an optical phase modulator. The phase modulated signal is filtered by a cavity (optical frequency reference) followed by a photodetector. The photodetected signal is amplified using a trans-impedance amplifier and down-converted by the same local oscillator frequency utilizing a frequency mixer. The down-converted signal is low pass filtered to generate the error signal. The detailed mathematical derivation of the PDH error signal has been published previously[3].

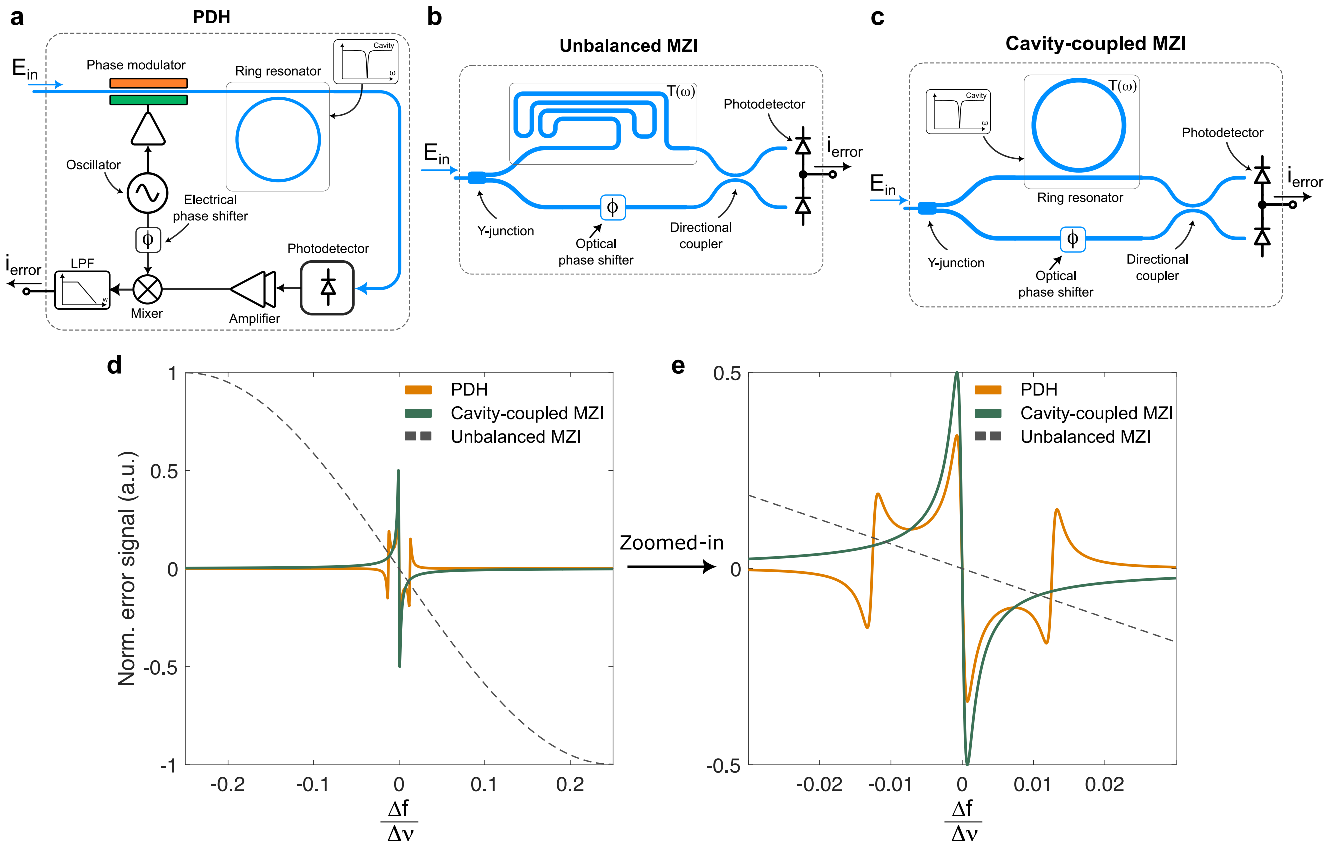

Supplementary Figure 2 | Comparison between the PDH, an unbalanced MZI, and the proposed cavity-coupled MZI OFNDs. **a** The block diagram of the PDH architecture. **b** The schematic of the unbalance MZI. **c** The schematic of the proposed cavity-coupled MZI. **d** and **e** are the simulated error signals of the aforementioned OFNDs. For this simulation, the circumference of the ring resonators in the PDH and cavity-coupled MZI schemes is identical to the waveguide length in conventional unbalanced MZI and is equal to 1 mm. The waveguide loss is assumed 0.2 dB.cm^{-1}.

Supplementary Figure 2b shows the schematic of an unbalanced MZI where the frequency reference is the length difference (true-time delay) in one arm of the MZI. Using Supplementary Equation (9) and substituting $T(\omega) = e^{j\omega\tau}$, we can conclude that the error signal is sinusoidal. Supplementary Figure 2c shows the block diagram of the proposed cavity-coupled MZI where the frequency reference is a microring resonator. Supplementary Figure 2d, e display the calculated error signal and the zoomed-in view of it, respectively. To ensure a fair comparison, given the same chip area, the circumference of the ring in both the PDH scheme and the cavity-coupled MZI configuration is set to 1 mm, which is equivalent to the length mismatch in the unbalanced MZI arrangement. For the purpose of this analysis, a propagation loss of 0.2 dB.cm^{-1} is assumed. Also, the local oscillator frequency in PDH architecture is 1 GHz, and the optical and electrical phase shifters (ϕ) are optimized accordingly. As shown in Supplementary Figure 2d, all three error signals are asymmetric around the reference frequency (f_{ref}). As suggested by Supplementary Figure 2d, the proposed cavity-coupled configurations has significantly more sensitivity compared to a conventional unbalanced MZI (K_{FD} =8×10^{-11} Hz^{-1}). As plotted in Supplementary Figure 2e, despite having a less complex design and potentially lower power consuming electronics, the proposed architecture exhibits a sensitivity (K_{FD} =1.7×10^{-8} Hz^{-1}) comparable to that of the PDH (K_{FD} =1.14×10^{-8} Hz^{-1}). It is important to highlight that in order to achieve the same OFND gain as the cavity-coupled MZI, the unbalanced MZI requires an equivalent delay of 17 nsec which is equivalent to approximately 1.27 m of TE-mode silicon waveguide. Implementing a delay line of such length on a silicon chip is impractical due to the limited available real estate on silicon and the associated waveguide loss. Furthermore, our proposed architecture eliminates the requirement for phase modulation, which may be unavailable in certain integrated photonic platforms or could introduce undesired residual amplitude modulation[4].

Supplementary Note 3: the OFND gain sensitivity analysis

The main building block in the proposed cavity-coupled MZI OFND is the frequency reference which is an on-chip high Q-factor microresonator. To achieve higher OFND gain, a microring resonator with a higher Q-factor and a large extinction ratio is required. The ring resonator coupling ratio (κ) and waveguide propagation loss (α) are the two crucial parameters that ultimately affect the OFND gain. Supplementary Figure 3 shows the simulation of the normalized OFND gain sensitivity to the ring resonator coupling and waveguide loss. In this simulation, a ring resonator with circumference of 1 mm, waveguide loss (α_0) of 0.2 dB.cm^{-1}, and critical coupling condition ($\kappa_0 = 0.5\%$) is considered. Maximum gain of OFND (K_{FD0}) happens at the lowest waveguide loss ($\alpha/\alpha_0 = 1$) and critical coupling condition ($\kappa/\kappa_0 = 1$), which in this scenario is about 1.7$\times$10^{-8} Hz^{-1}. Supplementary Figure 3 suggests that for operating close to optimum performance (i.e., $K_{FD}/K_{FD0} > 0.9$), it is necessary to have the waveguide loss within 10% of the nominal value. However, the coupling ratio margin in this case does not require such a stringent constraint. It is worth mentioning that while the fabricated chip may reside in lower performance regions due to fabrication-induced errors and inaccurate roughness estimation, it is noteworthy that the overall loop gain can be compensated for by electronic amplifiers.

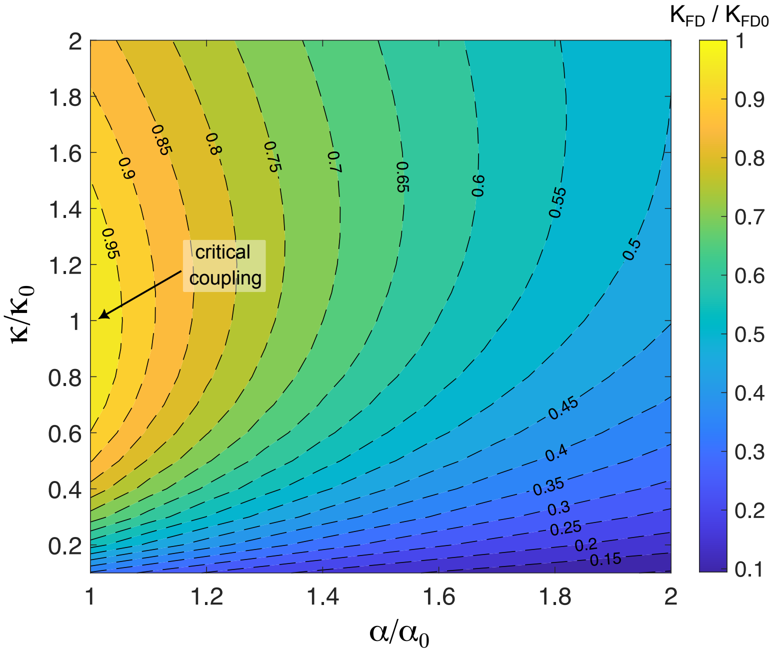

Supplementary Figure 3 | The OFND gain sensitivity analysis to the ring resonator coupling and waveguide loss. In this analysis, the OFND gain is normalized to the ideal scenario for a critically coupled ring resonator ($\kappa_0 = 0.5\%$) with circumference of 1 mm and waveguide loss α_0 of 0.2 dB.cm^{-1}. The maximum normalized gain (K_{FD0}) is 1.7$\times$10^{-8} Hz^{-1}.

Supplementary Note 4: Noise sources

Input referred noise of electronics

The input referred noise of an amplifier characterizes the noise level at the input of the amplifier and represents the equivalent noise voltage or current that, if applied directly to the input, would produce the same output noise. The input referred noise of electronics is a function different circuit variables such as temperature, circuit topology, and transistor technology. Assuming a chain of n amplifiers with gain G_i and input referred noise power spectral density (PSD) of N_i, using Friis equation[6], the total input referred noise can be written as

$$N_{total} = N_0 + \frac{N_1}{G_0^2} + \frac{N_2}{(G_0G_1)^2} + ... + \frac{N_{n-1}}{(G_0G_1...G_{n-2})^2}. \qquad (11)$$

As suggested by Supplementary Equation (11), the noise performance of the first stage is the most dominant noise contributor and, hence, a designer should consider highest gain and lowest noise for the first amplifier stage (G_0, N_0). Supplementary Figure 4a shows the circuit of the transimpedance amplifier (TIA) used in this work. Supplementary Figure 4b shows the frequency response of the TIA with gain of 5 kΩ and 3-dB bandwidth of about 10.8 MHz. Supplementary Figure 4c demonstrates the simulated input referred current noise of the TIA which is about 3.2 pA.Hz$^{-1/2}$. The input of the TIA is wirebonded to the output of the on-chip BPDs and its output is connected to the PID controller with input referred voltage noise of $<$5 nV.Hz$^{-1/2}$ which makes the total input referred current noise of electronics about 3.4 pA.Hz$^{-1/2}$.

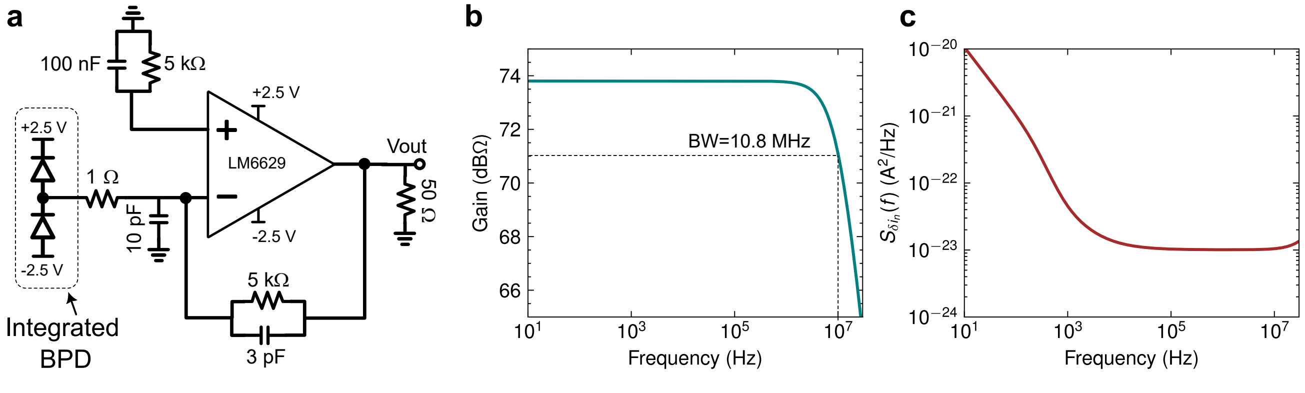

Supplementary Figure 4 | Transimpedance amplifier. a The architecture of the implemented transimpedance amplifier (TIA) using LM6629 operational amplifier. **b** The gain response of the TIA with 3-dB bandwidth (BW) of about 10.8 MHz. **c** The simulated PSD of the TIA input referred current noise.

Photodetector shot noise

The shot noise of a photodetector is frequency independent and its PSD is

$$S_{\delta i_{shot}}(f) = 2qI_0, \tag{12}$$

where q and I_0 are unit charge (*i.e.* $1.6 \times 10^{-19}C$) and DC photo-current, respectively. For a BPD arrangement, two photodetectors generate uncorrelated noise and hence the shot noise PSD is twice the PSD of each photodetector. Assuming 50 μW optical power at the input of the on-chip MZI structure, the total shot noise is about 4 pA.Hz$^{-1/2}$.

Laser intensity noise

In an electro-optic closed-loop system, laser intensity noise is another important source of noise. However, in the proposed system with its balanced detection architecture, laser intensity noise does not dominate. Let's assume the noise in the intensity of the laser is $\delta p(t)$. The electric field E_{in} in Supplementary Figure 1a can be written as

$$E_{in}(t) = \sqrt{P_0 + \delta p(t)}e^{j\omega t}, \tag{13}$$

where P_0 and ω are the average laser power and instantaneous frequency. As a result, Supplementary Equations (2) and (3) can be modified to

$$E_1(t) = \sqrt{\frac{P_0 + \delta p(t)}{2}}T(\omega)e^{jwt}, \tag{14}$$

$$E_2(t) = \sqrt{\frac{P_0 + \delta p(t)}{2}}e^{j\phi}e^{jwt}. \tag{15}$$

Using Supplementary Equations (4) to (7), the photo-currents of $i_1(t)$ and $i_2(t)$ are

$$i_1(t) = I_{cm} + R\Im(E_1E_2^*), \tag{16}$$

$$i_2(t) = I_{cm} - R\Im(E_1E_2^*), \tag{17}$$

where I_{cm} is the common mode current and can be written as

$$I_{cm} = \frac{RP_0}{4}(1 + \frac{\delta p(t)}{P_0})\left(1 + |T(\omega)|^2\right). \tag{18}$$

As can be seen from Supplementary Equation (18), the common mode photo-current, I_{cm} contains laser intensity noise. However, due to balanced detection architecture (Supplementary Figure 1a), the signal of interest (i.e., i_{error}) is generated by $i_1(t) - i_2(t)$. As a result, the common mode current and significant portion of the effect of laser intensity noise on the error signal is canceled. It is also worth mentioning that assuming the laser frequency is at optical cavity resonance (closed-loop condition) and MZI phase is biased properly ($\phi = k\pi$), then $|T(\omega)| \times \sin(\psi(\omega) - \phi)$ is zero and the contribution of laser intensity noise to the differential current is negligible too.

Thermo-refractive noise

Thermal noise of a nanophotonic waveguide

A silicon nanophotonic waveguide with thickness of 220 nm, width of 500 nm, and length of L is considered. The phase of optical signal propagating for a distance of L, changes by

$$\phi = \frac{\omega}{c} n_{eff}(\omega, T)L, \tag{19}$$

where ω, c, and $n_{eff}(\omega, T)$ are optical wave frequency, speed of light in vacuum, and the frequency and temperature dependent effective index. Any temperature fluctuation perturbs the index of refraction, hence introducing phase noise along the waveguide length which can be written as

$$\delta\phi = (\frac{\omega}{c} L \frac{\partial n_{eff}}{\partial T})\delta T, \tag{20}$$

$$S_{\delta\phi}(f) = (\frac{\omega}{c} L \frac{\partial n_{eff}}{\partial T})^2 \; S_{\delta T}(f), \tag{21}$$

where δT and $\delta\phi$ are temperature fluctuation and the random phase perturbation due to temperature change, respectively. $S_{\delta T}(f)$ and $S_{\delta\phi}(f)$ are PSDs of temperature noise and thermal induced phase noise, respectively. Using finite element waveguide mode solver, as shown in Supplementary Figure 5a, the thermo-optic coefficient of the effective index of TE_0 mode inside the silicon waveguide, $\frac{\partial n_{eff}}{\partial T}$, (at wavelength of 1550 nm) is about $1.94 \times 10^{-4} \; K^{-1}$. The thermal fluctuations satisfy thermal diffusion equation[7]

$$\gamma \nabla^2 \delta T = -\frac{\partial \delta T}{\partial t}, \tag{22}$$

where γ is thermal diffusivity. A general solution to Supplementary Equation (22) is

$$\delta T(t) = \delta T_0 e^{i\left(\omega_0 t - \bar{\beta}\cdot\bar{r}\right)}, \tag{23}$$

$$(\delta T_0)^2 = \frac{1}{(2\pi)^3}\frac{k_B T^2}{\rho C_\rho}, \tag{24}$$

where β, k_B, ρ, C_ρ, and ω_0 are mode [m^{-1}], Boltzmann constant [$J.K^{-1}$], material density [$kg.m^{-3}$], specific heat [$J.kg^{-1}$], and diffusion rate [$\omega_0(rad/s) = i\beta^2\gamma$], respectively. Using Supplementary Equation (23), the autocorrelation function can be written as

$$R(\tau) = (\delta T_0)^2 |I|^2 e^{-\beta^2\gamma|\tau|}, \tag{25}$$

$$I = \frac{1}{V}\iiint e^{-i\bar{\beta}.\bar{r}} d^3 r, \tag{26}$$

where $R(\tau)$ and V are the autocorrelation function and total volume of the medium.

The PSD of the thermal induced phase noise can be derived by using Supplementary Equation (21) and Fourier transform of Supplementary Equation (25). For a long waveguide, we can utilize cylindrical

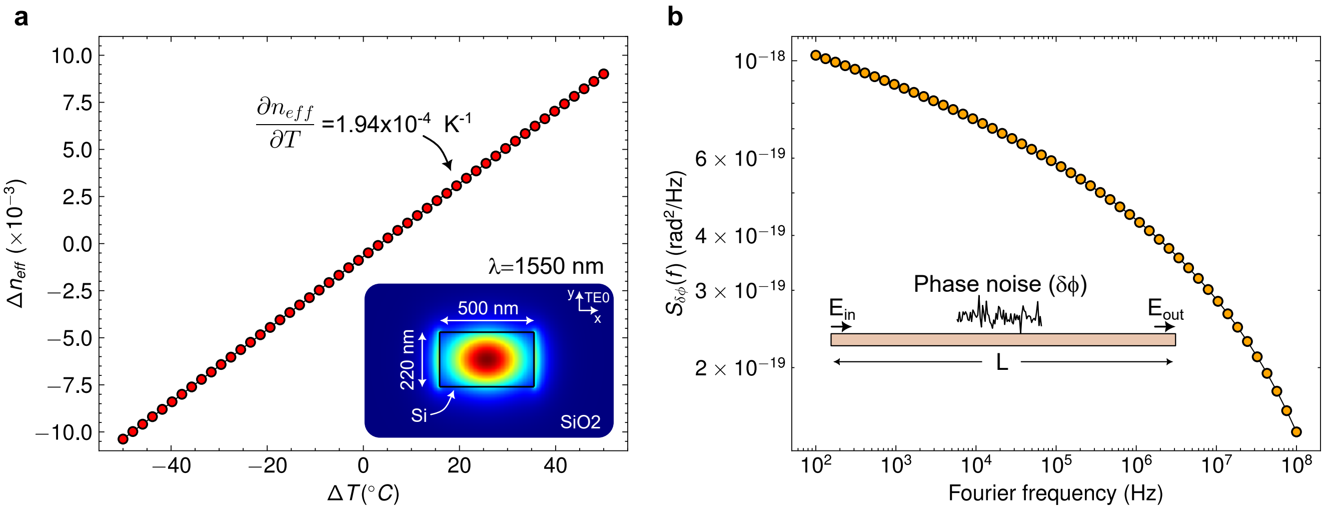

Supplementary Figure 5 | Thermal noise of a nanophotonic silicon waveguide. a The effective index change (Δn_{eff}) of the fundamental TE mode in a silicon waveguide as a function of temperature change. The optical mode is solved using finite element waveguide simulator. At $T_0 = 300K$, n_{eff} is approximately 2.4451 at wavelength of 1550 nm. **b** PSD of thermal induced phase noise in a $500\mu m$ long silicon waveguide.

coordinates and write the PSD of phase noise as[7]

$$S_{\delta\phi}(\omega) = S_0 \iint \frac{\sin^2[(\beta L/2)\cos\theta]}{\beta^2\cos^2\theta} \frac{J_1^2(\beta a \sin\theta)}{\beta^2 a^2 \sin^2\theta} \sin\theta d\theta \cdot \frac{\beta^4\gamma}{\omega^2 + (\beta^2\gamma)^2} d\beta, \tag{27}$$

$$S_0 = \frac{16}{\lambda^2\pi} \frac{k_B T^2}{\rho C_p} \left(\frac{\partial n_{eff}}{\partial T}\right)^2, \tag{28}$$

where L, λ, ω, and a are waveguide length, wavelength of light, Fourier frequency, and the effective radius of the waveguide cross section. In a waveguide with rectangular cross section, a can be calculated as $\sqrt{\frac{w\times d}{\pi}}$, where w and d are the waveguide width and thickness, respectively. Supplementary Figure 5a (inset) is the simulated TE$_0$ mode at $T_0 = 300K$ and wavelength of 1550 nm. Supplementary Figure 5b shows the PSD of the thermal induced phase noise for a silicon nanophotonic waveguide with length of $500\mu m$ and cross section shown in Supplementary Figure 5a (inset).

MZI noise due to waveguide TRN

As mentioned earlier, a waveguide with length of L at temperature T_0 will induce phase noise to the propagating optical wave due to thermo-refractive fluctuations. If such waveguide is used in an MZI arrangement followed by photodetector, the phase noise will translate into electronic amplitude noise. Consider Supplementary Figure 1a where the frequency reference is assumed noise-less (ideal) and the only noise source we consider is the thermo-refractive noise of the waveguide in the bottom branch which induces phase noise to E_2. Using Supplementary Equation (9), ϕ can be interpreted here as the

waveguide TRN. Any small perturbation in phase will perturb the error signal as

$$\frac{\partial i_{out}}{\partial \phi} = -RP_0|T(\omega_0)|\cos(\psi(\omega_0)), \tag{29}$$

where ω_0 is the laser frequency. Assuming the laser is locked to the resonance of the cavity, Supplementary Equation (29) is used to estimate the PSD of the induced electronic noise due to the waveguide TRN that is

$$S_{\delta i_n}(f) = (RP_0|T(\omega_0)|)^2 \times S_{\delta\phi}(f). \tag{30}$$

As an example, for $50\mu W$ input optical power to the MZI structure and ring resonator extinction ratio of about 5 dB, the estimated rms current noise induced by the TRN of a $500\mu m$ long silicon waveguide discussed in Supplementary Figure 5(b), is about 7.2 fA.Hz$^{-1/2}$. This is significantly lower than the photodetector shot noise and total input-referred noise of the TIA.

Microresonator TRN

The resonance frequency of a microring resonator is defined based on the round-trip phase inside the resonator. As a result, any phase perturbation such as TRN of the waveguide will perturb the resonance frequency of the resonator and, using similar approach discussed earlier, the perturbation of resonance frequency can be estimated. One of the models that has been developed to estimate the TRN of the micro-cavities assumes a homogeneous microresonator in an infinite heat bath[5,8]. The PSD of the random frequency fluctuations of the fundamental mode resonance can be written as[5]

$$S_{\delta f}(\omega) = \left(\frac{f_0}{n_{eff}}\frac{\partial n_{eff}}{\partial T}\right)^2 \frac{k_B T^2}{\sqrt{\pi^3 \kappa \rho C_p \omega}} \frac{1}{R\sqrt{d_x^2 - d_y^2}} \frac{1}{\left[1 + (\omega\tau_d)^{3/4}\right]^2}, \tag{31}$$

$$\tau_d = (\frac{\pi}{4})^{\frac{1}{3}} \frac{\rho C_p}{\kappa} d_x^2 \tag{32}$$

where $R, \kappa, \tau_d, f_0, dx,$ and dy are the effective radius of the microring, thermal conductivity, optical frequency, half-width of the fundamental mode at half-maximum of intensity with respect to x (width) and y (thickness) dimensions, respectively. While this theoretical model may lack precision, it provides valuable insights into engineering the TRN. As suggested by Supplementary Equation (31), the TRN of the cavity is a quadratic function of materials thermo-optic coefficient, $\frac{\partial n_{eff}}{\partial T}$. In other words, a waveguide medium with, ideally, zero or negligible thermo-optic coefficient is desired. Another important design parameter is the microresonator size. As suggested by the model, the PSD of TRN is inversely proportional to R. In the case of a ring resonator, this implies that as the mode volume ($\propto R$) increases, the TRN decreases. This theoretical model assumes a homogeneous microresonator in an infinite heat bath which may not be accurate for integrated microresonators with complex waveguide geometry and made from different

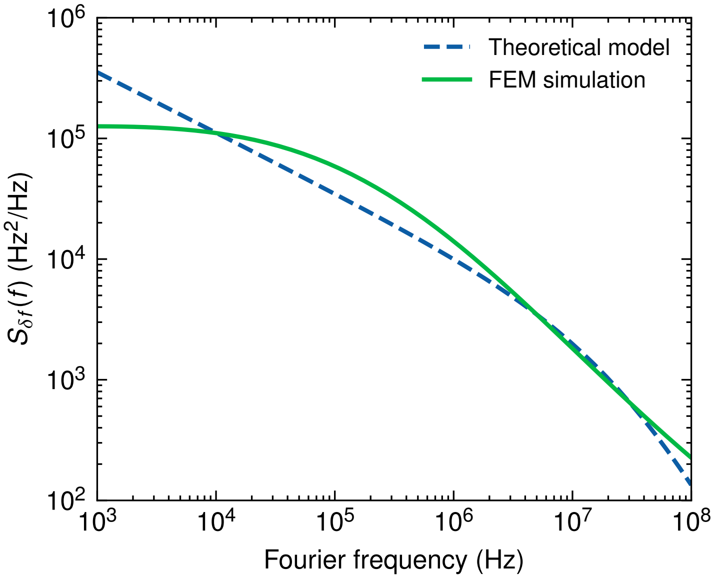

Supplementary Figure 6 | Thermo-refractive noise of a silicon microring resonator. The TRN noise of microring resonator described in the main manuscript is simulated with COMSOL FEM simulation (green) and compared with analytical model (blue).

materials. A finite element method (FEM) simulation that is based on fluctuation-dissipation theorem[8,9] can describe TRN behaviour of a microresonator more accurately.

Supplementary Figure 6 shows the TRN of the microring resonator used in this work, using the analytical model described in Supplementary Equation (31) and FEM simulation. It is clear that despite an overall agreement between the two methods, the theoretical model matches the FEM results better for higher Fourier frequencies. The low pass behaviour of the FEM is due to the limited geometry of the device which clearly is not the case for theoretical model that assumes an infinite heat bath. The details of COMSOL FEM simulation can be found in Ref.[5].

Supplementary Note 5: the closed-loop operation in presence of noise sources

Supplementary Figure 7 shows the simplified block diagram of the closed-loop laser frequency noise suppression. The control loop is linearized around the reference frequency (f_{ref}), in presence of different noise sources. As shown in Supplementary Figure 7, there are three dominant noise sources in the loop contributing to the closed-loop noise performance. These include laser intrinsic frequency noise (δf_n), the thermorefractive noise (TRN, δf_{TRN}) of the frequency reference used in the optical frequency noise discriminator (OFND), and the total electronics noise (δi_n) that includes input referred noise of electronics and shot noise of BPDs. The small signal current perturbation due to the frequency fluctuations is

$$\delta i_{error} = K_{FD} \times (\delta f_{laser} + \delta f_{TRN}), \tag{33}$$

where δf_{laser} and K_{FD} [A.Hz^{-1}] are laser frequency noise under closed-loop condition and conversion gain of OFND, respectively. A small change in the error signal is amplified by electronic gain, K_E [A.A^{-1}]. The total current noise at the input of the electronics, δi_n, includes the input-referred current noise of the amplifiers and shot noise of the BPD. The laser current perturbation is

$$\delta i_{laser} = K_E \times (\delta i_n + \delta i_{error}). \tag{34}$$

The small signal current modulates laser frequency by frequency modulation gain of K_L [Hz.A^{-1}],

$$\delta f_{error} = K_L \times \delta i_{laser}. \tag{35}$$

Under the closed-loop operation,

$$\delta f_{laser} = \delta f_n - \delta f_{error}. \tag{36}$$

Using Supplementary Equations (33) to (36) (assuming $K_E K_L K_{FD} >> 1$),

$$\delta f_{laser} \approx (\frac{1}{K_E K_L K_{FD}})\delta f_n - (\frac{1}{K_{FD}})\delta i_n - \delta f_{TRN}. \tag{37}$$

Since $\delta f_n, \delta i_n$, and δf_{TRN} are independent random variables with an average of zero, the PSD of the laser frequency noise is

$$S_{laser}(f) \approx (\frac{1}{K_E K_L K_{FD}})^2 S_0(f) + (\frac{1}{K_{FD}})^2 S_n(f) + S_{TRN}(f), \tag{38}$$

where $S_{laser}(f), S_0(f), S_n(f)$, and $S_{TRN}(f)$ are the power spectral densities of stabilized laser frequency noise, free-running laser frequency noise, electronic noise, and the cavity TRN, respectively. As suggested by Supplementary Equation (38), the PSD of the free-running laser is suppressed by the open loop gain. It is worth noting that utilizing a sensitive OFND (i.e. high K_{FD}) can significantly enhance frequency noise

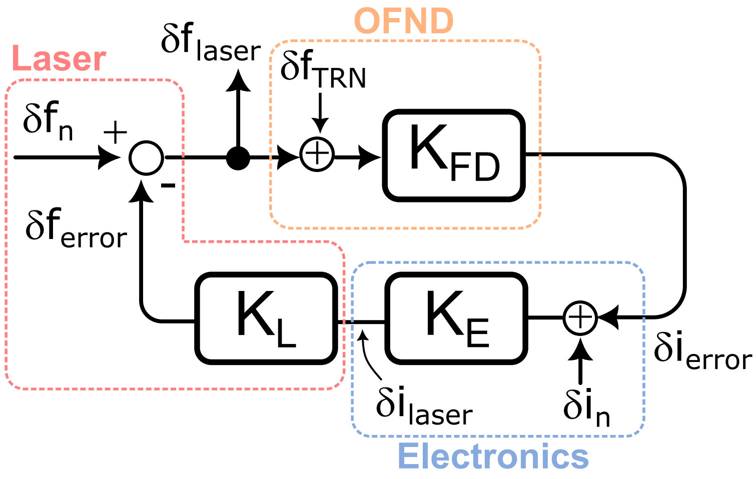

Supplementary Figure 7 | The linearized closed-loop block diagram. $\delta i_n, \delta f_n$, and δf_{TRN} are input referred noise of electronics, intrinsic frequency noise of laser, and thermorefractive noise of the cavity, respectively.

detection gain and enhance the frequency noise suppression. Moreover, the ultimate laser frequency noise under closed-loop operation is limited by

$$S_{limit}(f) = (\frac{1}{K_{FD}})^2 S_n(f) + S_{TRN}(f). \tag{39}$$

According to Supplementary Equation (39), the TRN of the cavity can impose a limit on the achievable frequency stability. Therefore, considering the specific requirements of an application, the ultimate achievable frequency noise can be engineered by a careful design and proper selection of the material platform for implementing the frequency reference[5]. Details of cavity TRN calculation is discussed in Supplementary Note 4.

Example: a laser locked to a high-Q factor silicon nitride resonator

As mentioned in the discussion section of main manuscript, our proposed cavity-coupled MZI laser frequency stabilization can be applied in various platforms. In this example, the optical frequency reference is made from low loss silicon nitride and the goal is to estimate the frequency noise and linewidth of the a laser locked to this reference using the proposed frequency locking technique.

For the sake of argument, the waveguide cross-section of 2.1μm $\times$ 0.9μm is assumed with optical propagation loss of 1 dB.m^{-1} as mentioned in Ref.[10]. Using model parameters in Supplementary Table 1, the critically coupled SiN microring resonator has a Q-factor of 2.3$\times$10^7 and a circumference of approximately 0.47 m corresponding to FSR of 250 MHz. Supplementary Equation (9) is used to calculate the OFND frequency discrimination gain which is about 2.3$\times$10^{-11} A.Hz^{-1} (Supplementary Figure 8a). Given the simulated mode profile and the resonator geometry, Supplementary Equation (31) is used to estimate the microresonator TRN. Without loss of generality, we consider a free-running laser with

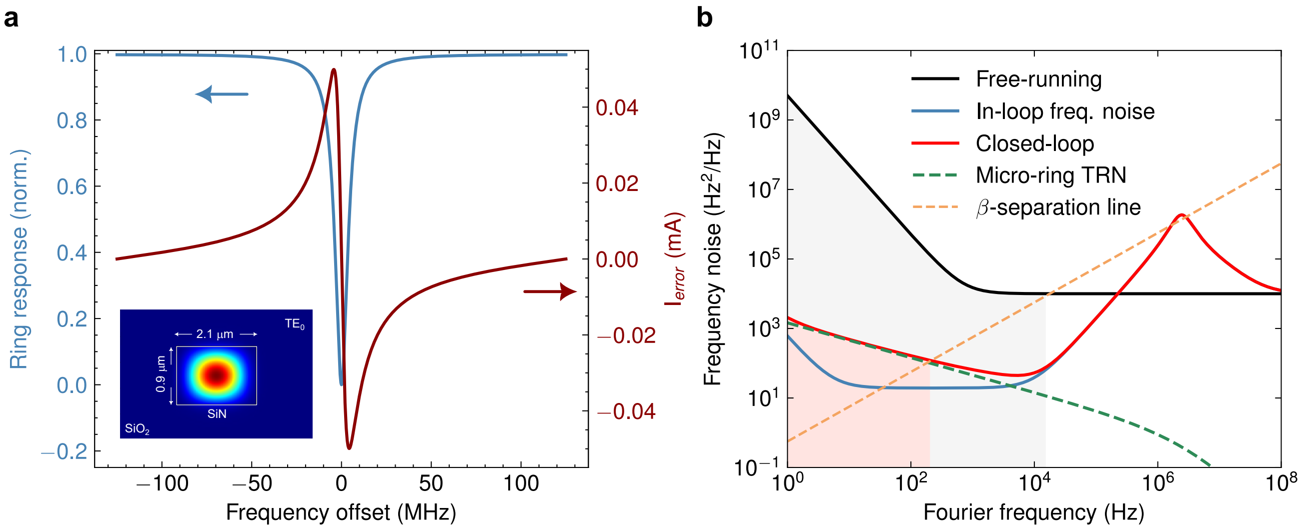

Supplementary Figure 8 | Simulation of the frequency noise PSD of a laser locked to a SiN microring. **a** The normalized rig response (blue) of the silicon nitride (SiN) microresonator. The error signal (red), I_{error}, is asymmetric around the ring resonance. **b** The free-running and stabilized laser frequency noise PSD.

Lorentzian linewidth of about 30 kHz and frequency noise PSD shown in Supplementary Figure 8b. The PSD of the stabilized laser frequency noise is then calculated based on Supplementary Equation (38). As demonstrated in Supplementary Figure 8b, the cavity TRN of the silicon nitride resonator in this example is significantly lower than that of the implemented silicon ring shown in the main manuscript. With that said, the combination of a higher servo-loop bandwidth and a lower cavity TRN is expected to result in a substantial reduction in the integral linewidth of the laser. The highlighted regions, distinguished by the β-separation line in Supplementary Figure 8b, indicate that the free-running integral linewidth of the laser is suppressed from 170 kHz to 480 Hz which corresponds to linewidth reduction factor of about 354.

Supplementary Note 6: Design procedure and considerations:

As mentioned earlier, the proposed laser frequency locking architecture is not bound to a specific platform and can be implemented in a technology that suits the designer's application. Here, a design procedure and considerations for implementation of such laser frequency locking technique in an integrated photonic platform are provided.

- Material platform: Choice of the host material in which the photonic integrated circuit (PIC) is implemented, depends on few factors. First, given the desired wavelength of operation, the waveguide material has to have low material absorption. Other available photonic components in that platform have to support that wavelength too. Among the available PIC fabrication technologies, the designer should choose the one that provides nanophotonic waveguides with the lowest optical propagation loss to achieve high-Q factor resonators.

- Target linewidth and chip area: The available chip area and cost are another important parameters need to be taken into account during the design. This will limit the size of the photonic components, including the optical frequency reference. As mentioned earlier, there is a trade-off between the size of the optical frequency reference and the minimum achievable TRN limit. Given the chip area, required TRN, and the propagation loss of the waveguide, the Q-factor of the resonator can be optimized.

- Noise analysis: For an effective link budget analysis, it is crucial to comprehend and accurately estimate the contributions of different noise sources. Different noise sources that may have significant contribution are cavity TRN, photodetector shot noise, and total input referred noise of electronics.

- Link budget analysis: an open loop simulation with estimated waveguide propagation loss gives the estimated OFN gain. To achieve stabilization for a specific laser, it's important to factor in optical power, laser frequency modulation gain, and total optical insertion loss during the link budget analysis. To attain the desired loop gain, bandwidth, and noise levels, the electronics, including TIA, amplifiers, and filters, must be carefully designed based on the gain and noise level of other sub-systems in the loop.

Supplementary Table

Parameter	Value	Parameter	Value
Microresonator FSR	250 MHz	Laser FM response[11]	b=2, fc=1.6 MHz
Optical loss	1 dB.m^{-1}	Input referred current noise	3.2 pA.Hz$^{-1/2}$
Laser power	100 μW	Laser current driver gain and BW	2 mA.V^{-1}, 10 kHz
n$_g$	2.56	Density (ρ)	3.29$\times$10^2 kg.m^{-3}
n$_{eff}$	2.34	Thermo-optic coeff.($\partial n/\partial T$)	2.45$\times$10^{-5} K^{-1}
Laser FM gain	500 MHz.mA^{-1}	Thermal conductivity	30 W.m^{-1}.K^{-1}
Servo loop gain, BW	125 kΩ, 1 MHz	Specific heat capacity	800 J.kg^{-1}.K^{-1}

Supplementary Table 1 list of model parameters used for the closed-loop simulation.

Equipment	Model
DFB laser 1	AeroDiode 1550LD-2-0-0-1
DFB laser 2	Gooch & Housego-AA1401
DFB laser 3	AeroDiode 1550LD-6-0-0-1
Tunable ECDL laser	TOPTICA CTL 1550
Reference laser	OEwaves OE4028
Frequency comb	Vescent FFC-100
Photodetector	LAB BUDDY DSC30S-39
Laser current source	LDX-3620B
Temperature controller	LDT-5910B, WTC3243
Oscilloscope	SDS1204X-E
Waveform generator	Keysight 33600A
Power supply	SIGLENT SPD3303X-E
FPGA	Redpitaya STEMlab 125-14
PID controller	Vescent D2-125
Optical filter	Santec OTF-350
Optical spectrum analyzer	YOKOGAWA AQ6370D

Supplementary Table 2 | List of equipment and components used in different experimental setups.

References

1. Drever, R. W. *et al.* Laser phase and frequency stabilization using an optical resonator. *Appl. Phys. B* **31**, 97–105 (1983).
2. Idjadi, M. H. & Blanco-Redondo, A. Enhanced frequency noise discrimination using cavity-coupled mach-zehnder interferometer. *arXiv preprint arXiv:2305.10525* (2023).
3. Black, E. D. An introduction to pound–drever–hall laser frequency stabilization. *Am. journal physics* **69**, 79–87 (2001).
4. Shen, H., Li, L., Bi, J., Wang, J. & Chen, L. Systematic and quantitative analysis of residual amplitude modulation in pound-drever-hall frequency stabilization. *Phys. Rev. A* **92**, 063809 (2015).
5. Huang, G. *et al.* Thermorefractive noise in silicon-nitride microresonators. *Phys. Rev. A* **99**, 061801 (2019).
6. Friis, H. T. Noise figures of radio receivers. *Proc. IRE* **32**, 419–422 (1944).
7. Glenn, W. H. Noise in interferometric optical systems: An optical nyquist theorem. *IEEE journal quantum electronics* **25**, 1218–1224 (1989).
8. Kondratiev, N. & Gorodetsky, M. Thermorefractive noise in whispering gallery mode microresonators: Analytical results and numerical simulation. *Phys. Lett. A* **382**, 2265–2268 (2018).
9. Weng, W., Light, P. S. & Luiten, A. N. Ultra-sensitive lithium niobate thermometer based on a dual-resonant whispering-gallery-mode cavity. *Opt. letters* **43**, 1415–1418 (2018).
10. Liu, J., Huang, G., Wang, R., He, J., Raja, A., Liu, T., Engelsen, N. & Kippenberg, T. High-yield, wafer-scale fabrication of ultralow-loss, dispersion-engineered silicon nitride photonic circuits. *Nature Communications*. **12**, 2236 (2021)
11. Corrc, P., Girad, O. & Faria, I. On the thermal contribution to the FM response of DFB lasers: Theory and experiment. *IEEE Journal Of Quantum Electronics*. **30**, 2485-2490 (1994)
